# Surface-modified engineered exosomes attenuated cerebral ischemia/reperfusion injury by targeting the delivery of quercetin towards impaired neurons

**DOI:** 10.1186/s12951-021-00879-4

**Published:** 2021-05-17

**Authors:** Lin Guo, Zhixuan Huang, Lijuan Huang, Jia Liang, Peng Wang, Liang Zhao, Yijie Shi

**Affiliations:** 1 School of Pharmacy, Jinzhou Medical University, Jinzhou, 121000 People’s Republic of China; 2 Life Science Institution, Jinzhou Medical University, Jinzhou, 121000 People’s Republic of China; 3 Key Laboratory of Neurodegenerative Diseases of Liaoning Province, Jinzhou Medical University, Jinzhou, 121000 People’s Republic of China

**Keywords:** ROS, Ischemia, Quercetin, Exosomes, GAP43

## Abstract

**Background:**

The incidence of ischemic stroke in the context of vascular disease is high, and the expression of growth-associated protein-43 (GAP43) increases when neurons are damaged or stimulated, especially in a rat model of middle cerebral artery occlusion/reperfusion (MCAO/R).

**Experimental:**

**design:**

We bioengineered neuron-targeting exosomes (Exo) conjugated to a monoclonal antibody against GAP43 (mAb GAP43) to promote the targeted delivery of quercetin (Que) to ischemic neurons with high GAP43 expression and investigated the ability of Exo to treat cerebral ischemia by scavenging reactive oxygen species (ROS).

**Results:**

Our results suggested that Que loaded mAb GAP43 conjugated exosomes (Que/mAb GAP43-Exo) can specifically target damaged neurons through the interaction between Exo-delivered mAb GAP43 and GAP43 expressed in damaged neurons and improve survival of neurons by inhibiting ROS production through the activation of the Nrf2/HO-1 pathway. The brain infarct volume is smaller, and neurological recovery is more markedly improved following Que/mAb GAP43-Exo treatment than following free Que or Que-carrying exosome (Que-Exo) treatment in a rat induced by MCAO/R.

**Conclusions:**

Que/mAb GAP43-Exo may serve a promising dual targeting and therapeutic drug delivery system for alleviating cerebral ischemia/reperfusion injury. 
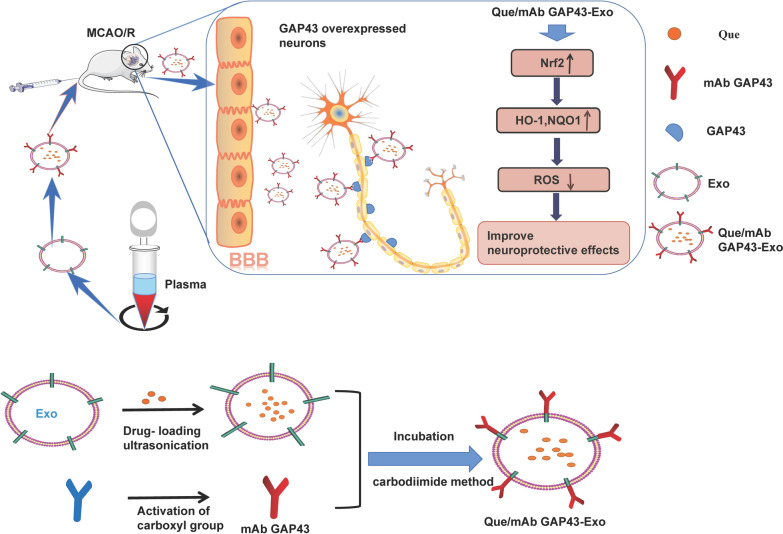

**Supplementary Information:**

The online version contains supplementary material available at 10.1186/s12951-021-00879-4.

## Background

Ischemic stroke is caused by ischemic necrosis of brain tissue due to insufficient cerebral blood flow, and the incidence of ischemic stroke is high in the context of vascular diseases [1, 2]. Long-term blood occlusion results in irreversible neuronal damage to neurons, and most patients suffer consequent language dysfunction and movement disorders. Although the United States Food and Drug Administration (FDA) proved that recombinant tissue plasminogen activator (tPA) is applied for the recanalization of the blocked blood vessels in the treatment of ischemic stroke, the therapeutic window of effective tPA-mediated thrombolysis is short (4.5 h), and 80% of patients experience serious sequelae or even death because optimal thrombolytic therapies are lacking [3–5]. Furthermore, thrombolytic therapy also increases the risk for patients with intracranial hemorrhage. Therefore, it is important to identify suitable and effective therapeutic strategies to alleviate cerebral ischemia.

Following the onset of ischemia/reperfusion, significant oxidative stress and inflammation are significantly triggered; subsequently, a large amount of reactive oxygen species (ROS) is produced in brain tissues, leading to neuronal injury via oxidation and destruction of neurons, thus aggravating severe brain damage and cerebral infarction [6, 7]. Nuclear factor erythroid-2-related factor 2 (Nrf2) is a critical antioxidative transcription factor that is a new target for scavenging ROS production and alleviates ROS-induced ischemia/reperfusion injury. It promotes the transcription of various antioxidant genes and contributes to maintaining redox homeostasis in cells [8, 9]. There is increasing evidence that the activation of Nrf2 and its downstream target genes can protect brain tissues from ischemia perfusion injury by inhibiting oxidative stress [10, 11].

Quercetin (Que; molecular formula: C_15_H_10_O_7_), a chemical found naturally in a number of foods, is a natural flavonoid polyphenol that exerts antioxidant effects by neutralizing free radicals, chemical byproducts that harm cell membranes and damage DNA [12, 13]. Que also reportedly plays a neuroprotective role by scavenging free radicals and exerting anti-inflammatory effects in cerebral ischemia models. Que can activate the Nrf2 pathway, and then promote the expression of detoxification enzymes and antioxidant enzymes, such as NAD(P)H: quinone oxidoreductase-1 (NQO1) and heme oxygenase-1 (HO-1), thereby inhibiting the production of a large amount of ROS and decreasing oxidative stress to normal levels in the ischemic brain tissue [14, 15]. However, its water insolubility, poor stability and inability to cross the blood–brain barrier (BBB) limit the ability of Que to treat central nervous system (CNS) diseases [16].

Drug delivery nanotechnologies have recently received significant attention due to their ability to improve the solubility and targeting specificity of drugs. Currently, therapeutic drug carriers are modified with specific recognizable ligands to achieve targeted drug delivery. Researchers have found that some aptamers such as chemical agents [17], antibodies [18] and cell-penetrating peptides (CPPs) [19, 20] can be modified into carriers to achieve targeted therapeutic effects with high specificity, selectivity and affinity. Exosomes derived from cells are nano-sized and saucer shaped, have low immunogenicity and are highly compatible. As exogenous substances such as chemicals, proteins, and nucleic acids can be efficiently loaded into exosomes by electroporation and incubation methods, exosomes potentiate a promising targeted drug delivery system for delivering drugs to specific regions [21–23]. However, naïve exosomes only target specific organs owing to their inherited homing and migration characteristics, thus reducing their ability to deliver substances to non-target organs and limiting their clinical application for some diseases, especially in CNS diseases. Some chemicals, proteins and antibodies have reportedly been modified on the surface of exosomes to construct a targeted, multifunctional, biological drug delivery system for enhanced therapeutics [24–26].

**Fig. 1:**
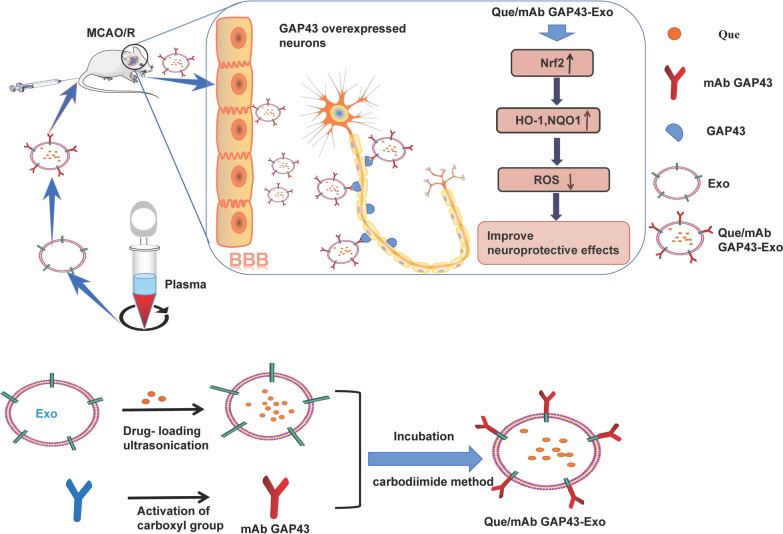
The primary hypothesis of this study. Exo were obtained from the whole blood of Sprague Dawley rats and encapsulated Que by ultrasonication. Finally, mAb GAP43 was modified on the surface of Que-loaded Exo using a carbodiimide method to obtain Que/mAb GAP43-Exo. Que/mAb GAP43-Exo enhanced the ability of Que to target the impaired, GAP43-overexpressed neurons. Furthermore, Que/mAb GAP43-Exo alleviated ischemia–reperfusion injury by inhibiting the production of ROS through the activation of the Nrf2/HO-1 pathway in a rat model of MCAO/R

Duo to the antioxidant effects of Que and the inherent cargo-carrying capacity of exosomes, we prepared Que-loaded exosomes (Que-Exo) to enhance the stability and solubility of Que. We also took advantage of the high expression of growth-associated protein-43 (GAP43) in damaged neurons as a potential guide to target impaired neurons. It has been reported that GAP43 is a neuron-specific protein and is involved in the growth of nerve cells and axonal development, as well as learning and memory functions [27, 28]. The protein expression of GAP43 increases when neurons are damaged or stimulated. In particular, in a MCAO/R animal model, GAP43 expression is increased in impaired neurons in ischemic penumbra [29, 30]. In our study, a monoclonal antibody against GAP43 (mAb GAP43) was modified on the surface of drug-loaded exosomes to target impaired neurons with a high expression of GAP43 in the ischemic penumbra to improve cerebral ischemia therapy. As shown in Fig. 1, exosomes (Exo) were isolated from the blood of Sprague Dawley (SD) rats, and Que was further loaded into Exo by ultrasonication. Finally, mAb GAP43 was conjugated to the surface of Que-Exo using the carbodiimide method to obtain Que/mAb GAP43-Exo. Given the interaction between Exo-delivered mAb GAP43 and GAP43 expressed in damaged neurons, Que/mAb GAP43-Exo enhanced drug accumulation by effectively delivering Que into GAP43-overexpressed injured neurons in ischemic penumbra and promoted neuron survival by inhibiting oxidative stress through the activation of the Nrf2/HO-1 pathway. Furthermore, compared with Que, Que/mAb GAP43-Exo significantly alleviated ischemic damage in brain tissues by reducing the infarct area and improving neurological performance. Taken together, these results suggested that Que/mAb GAP43-Exo effectively protected against cerebral ischemia and enhanced the neuroprotective effect of Que by neuron-targeted drug delivery.

## Materials and methods

### Materials

Que was purchased from Aladdin Chemical (Shanghai, China). All other reagents were obtained from Sigma-Aldrich Co (St Louis, MO, USA). The mAb GAP43 was purchased from Cell Signaling Technologies (Danvers, MA, USA). Male SD rats weighing 250–300 g were provided by the Animal Center of Jinzhou Medical University, and the animal protocols were approved by the Animal Protection and Use Committee of Jinzhou Medical University.

### Preparation of Que/mAb GAP43-Exo

Exo was isolated and purified from the whole blood of SD rats by gradient centrifugation. According to previous reports [31–33], Que was incubated with Exo in a medium comprising phosphate buffered saline (PBS), dimethyl sulfoxide (DMSO) and Tween 80 (85:10:5, v/v/v) under continuous ultrasonic shaking in an ice water bath to encapsulate Que in Exo. After centrifugation and PBS cleaning, the Que-Exo were obtained. The mAb GAP43 was modified on the surface of Que-Exo using a carbodiimide method. Ethyl-3-(3-dimethylaminopropyl) carbodiimide hydrochloride (EDC) was used to activate the carboxyl group on mAb GAP43, and then N-hydroxysuccinimide (NHS) was added to stabilize the intermediate. Finally, the activated carboxyl group of mAb GAP43 was combined with the amino group of Exo to obtain Que/mAb GAP43-Exo. The morphology and particle size of Que/mAb GAP43-Exo were assessed by atomic force microscopy (AFM) and a Zetasizer Nano ZS instrument. The loading ratio and encapsulation rate of Que in Que/mAb GAP43-Exo were estimated using a previously reported method [34].

### Assessment of solubility and stability in vitro

To evaluate the solubility of Que, 1 mg of Que was separately dispersed in 1 mL of PBS solution (pH 7.4) and 1 mL of mAb GAP43-Exo solution at 10 mg/mL by ultrasonic shaking and balancing for obtaining the saturated drug solution*.* After centrifugation at 12,000 rpm, undissolved Que were precipitated at the bottom of tube and the concentration of free Que in the supernatant was studied by dissolving Que with additional 4 times volume of methanol and measuring solubility of Que using high-performance liquid chromatography (HPLC). Next, Que dissolving in the supernatant in the presence of in mAb GAP43-Exo was also treated with 4 times volume of methanol to break lipid layer of Exo and dissolve Que completely. Finally, concentration of Que was measured to calculate the solubility of Que using HPLC. The chromatographic system included a Hypersil C18 column, a mobile phase containing methanol and phosphoric acid in water (pH 7.4; 0.01 M; 60:40, v/v), at a flow rate of 1.0 mL/minute and column temperature of 30℃, and the UV detection wavelength was set at 374 nm.

To evaluate the stability of Que in PBS and plasma, the oxidation and degradation of Que were studied by placing Que and Que/mAb GAP43-Exo in PBS and plasma to determine the UV absorbance of Que in the preparation. We selected a defined wavelength (374 nm) to detect the absorption value of Que in PBS and plasma. A certain amount of free Que and Que/mAb GAP43-Exo was dissolved in PBS and plasma. At different time intervals, methanol was added to extract Que. The UV absorbance of the mixture of blank PBS or plasma and methanol was set as the control group, and the UV absorbance of free Que and exosomal Que was valued at 374 nm. The remaining ratios (RR) of Que or exosomal Que were determined by calculating the ratio of the UV absorbance of Que or exosomal Que in PBS and plasma at a predetermined time to UV absorbance of Que or exosomal Que at the beginning, as based on the methods described in our previous report [35].

To assess drug release, Que and Que/mAb GAP43-Exo dispersed in PBS were added into a dialysis bag and immersed in a flask containing 100 mL of release medium (PBS, pH 7.4, 37 °C) containing 0.5% (w/v) Tween 80. Samples were taken at certain time points, and the amount of released Que was analyzed by HPLC.

### Biodistribution of Que in organs in vivo

SD rats were randomly divided into two groups and housed under thermoregulated, humidity-controlled conditions. Free Que and Que/mAb GAP43-Exo were dissolved in a mixture of PBS, DMSO, and Tween 80 (85:10:5, v/v/v) to achieve a Que concentration of 3.4 mg/mL and administered by tail vein injection. After 8 h of treatment, the accumulation of free Que and exosomal Que in different organ tissues was determined by HPLC.

### Administration of formulations in MCAO/R

To establish the MCAO/R model, a 6–0 nylon monofilament suture was inserted into the right internal carotid artery of each SD rat for 2 h and subsequently removed to allow blood reperfusion. PBS, Que, Exo, Que-Exo, and Que/mAb GAP43-Exo were administered to MCAO/R rats by intravenous (I.V.) injection via the tail vein.

### Differences in the distribution of Que and Que/mAb GAP43-Exo in vivo and in vitro

To further explore the GAP43-mediated targeting of damaged neurons by Exo, Exo and mAb GAP43-conjugated Exo (mAb GAP43-Exo) were labeled with red fluorescent PKH26 as described in our previous report [31], and confocal laser scanning microscopy was utilized to determine the localization of Exo in cells. Human neuroblastoma cells, SH-SY5Y cells were purchased from the Type Culture Collection of Chinese Academy of Sciences (Shanghai, People’s Republic of China) and cultured in Dulbecco's Modified Eagle Medium: Nutrient Mixture F-12 (DMEM/F-12) supplemented with 10% fetal bovine serum (FBS) and 1% PS. SH-SY5Y cells were seeded in six-well flat-bottom plates and subjected to oxygen–glucose deprivation injury (OGD) for 1 h according to the previous report [36, 37]. Finally, PKH26-labeled mAb GAP43-Exo was incubated with normal SH-SY5Y cells and OGD-treated SH-SY5Y cells; the uptake of mAb GAP43-Exo in both cells was determined by flow cytometry. To confirm the role of GAP43 in mediating the uptake of Exo, PKH26-labeled Exo and mAb GAP43-Exo were incubated with OGD-treated SH-SY5Y cells in the presence or absence of mAb GAP43. The cellular distributions of Exo and mAb GAP43-Exo were observed by detecting the fluorescence of PKH26 (Ex:551 nm, Em: 567 nm) using confocal laser scanning microscopy at predetermined intervals. To monitor the distribution of Que in the ischemic brain, Que, Que-Exo and Que/mAb GAP43-Exo were administered to MCAO/R rats by intravenous injection, and fluorescence images of Que (Ex: 436 nm and Em: 486 nm) of the brain were captured.

### Infarct measurement and neurological evaluation

2,3,5‐Triphenyltetrazolium chloride (TTC) was used to stain infarct tissues. The infarct volume percentage was determined and a neurological evaluation was performed by assessing the neurological function score.

### Oxidative stress measurement

According to our previous report [31], ROS production in ischemic brain tissues was detected by treating frozen section of brain tissue with 10 μM of 2',7'-dichlorodihydrofluorescein diacetate (DCFH-DA; Sigma-Aldrich Co) and observing the fluorescence of DCF (Ex: 485 nm, Em: 530 nm).

### LDH assay

Que, Que-Exo and Que/mAb GAP43-Exo were added into the plates and incubated with OGD-treated SH-SY5Y cells for 24 h. A lactate dehydrogenase (LDH) assay kit was used to measure the LDH content according to the protocol.

### Western blot analysis

SD rats were divided into the MCAO/R, Que, Que-Exo, Exo and Que/mAb GAP43-Exo groups. The ischemic penumbra was collected after administration and homogenized to prepare the sample. After being treated with electrophoresis, the proteins were transferred to polyvinylidene fluoride (PVDF) membranes and blocked with bovine serum albumin. The membranes were incubated with different kinds of primary antibodies overnight at 4 °C. After washing, the membranes were further incubated with a secondary antibody for 1 h at room temperature. Finally, the signal was visualized using enhanced chemiluminescence (iBox Scientia 600; UVP, LLC, Upland, CA, USA).

## Results

### Characterization of Que/mAb GAP43-Exo

**Fig. 2:**
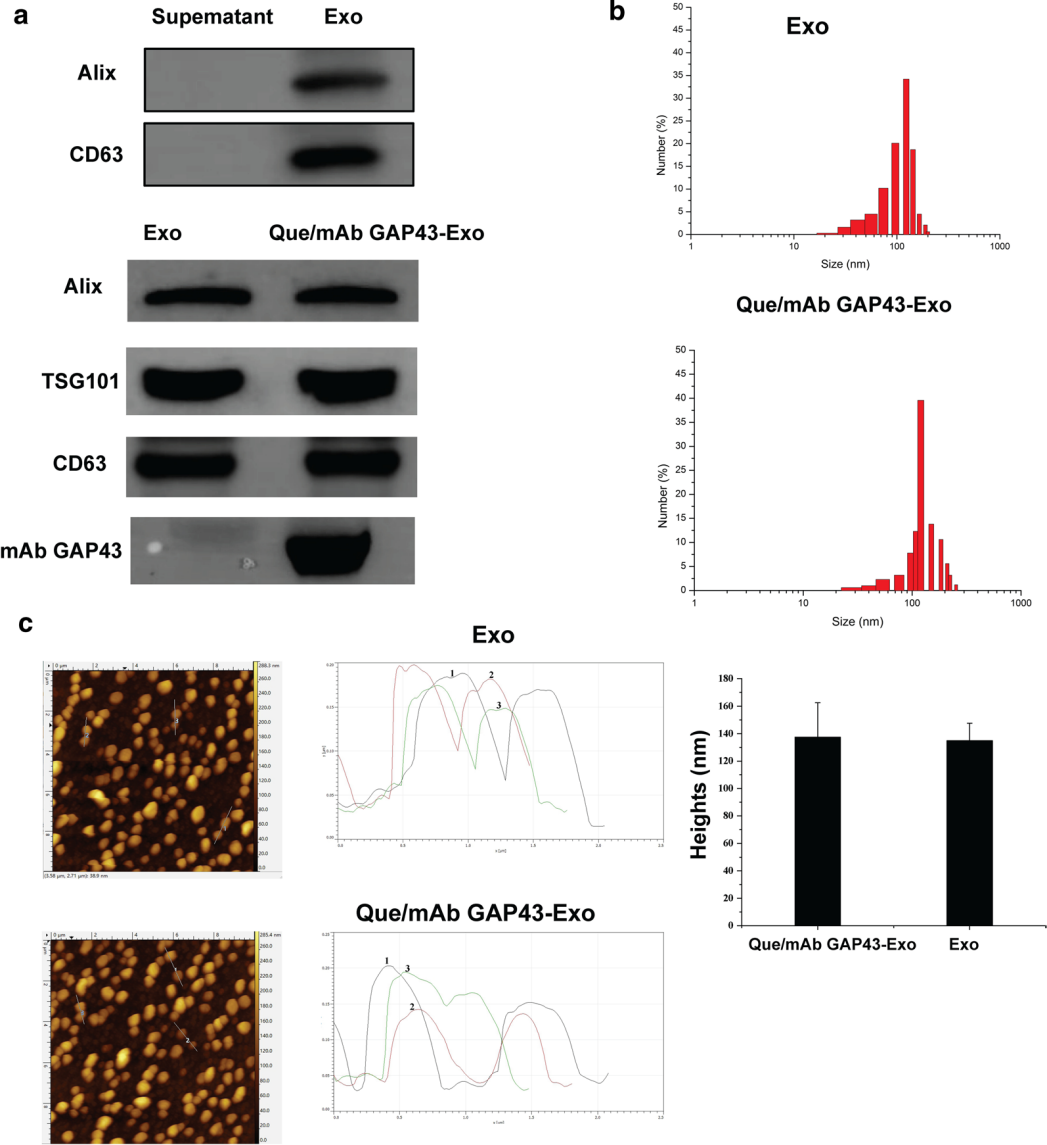
Characterization of Exo and Que/mAb GAP43-Exo. **a** Analysis of exosomes markers and mAb GAP43 in Exo and Que/mAb GAP43-Exo. **b** Particle size distribution of Exo and Que/mAb GAP43-Exo measured by Zetasizer Nano ZS. **c** Morphology of Exo and Que/mAb GAP43-Exo observed by AFM. The average heights of Exo and Que/mAb GAP43-Exo were analyzed. Data are expressed as means ± SD (n = 6)

We characterized Exo and Que/mAb GAP43-Exo by AFM, particle size analysis and Western blotting. Using AFM, a mechanical probe was used to measure the size and structure of individual extracellular vesicles in their native state. The Exo and Que/mAb GAP43-Exo were then placed on a freshly cleaved mica surface and then scanned. The AFM results (Fig. 2c) showed that Exo and Que/mAb GAP43-Exo were very broadly distributed and were small and spherical. We performed a depth analysis of the AFM data. As shown in the Fig. 2c, the heights of Exo and Que/mAb GAP43-Exo were calculated as the maximum height. The figure demonstrated the changes occurred to the exosomal height, representing the main morphological parameters. As illustrated, the heights of Exo and Que/mAb GAP43-Exo were about 100 nm and the average heights of Exo and Que/mAb GAP43-Exo were about 135.1 nm and 137.5 nm, indicating that there were no significant difference in height between Exo and Que/mAb GAP43-Exo. The results of the nanoparticle size analysis (Fig. 2b) also showed the average size respectively. Plasma was centrifuged, the bottom layer was collected to obtain Exo, and the residual supernatant, which contained no Exo, was used as the negative control. The Western blot results (Fig. 2a) showed that Exo was enriched with Alix and CD63, but that neither protein was expressed in the supernatant, indicating that all Exo had been completely isolated and collected from the plasma. Furthermore, both Exo and Que/mAb GAP43-Exo expressed common exosomal proteins, such as Alix, CD63, and TSG101 proteins [38]. All data indicated that the characterizatics of Exo did not change upon modification with Que and mAb GAP43. The presence of mAb GAP43 in Que/mAb GAP43-Exo was assessed by incubating Exo and Que/mAb GAP43-Exo with HRP-Rabbit antibody directly for 1 h, RT and detected by enhanced chemiluminescence (ECL). The mAb GAP43, which was expressed by Que/mAb GAP43-Exo, was not detected in Exo, indicating that mAb GAP43 was successfully conjugated to the surface of Exo. We also obtained the encapsulation efficiency of Que of 34.0% and a drug-loading efficiency of Que of 13.6%. Release analysis results (Additional file 1) demonstrated that the free Que was released faster than Que/mAb GAP43-Exo and that approximately 84.1% of total drug was released into the medium within the first 6 h. In contrast, Que/mAb GAP43-Exo demonstrated a steady and sustained release pattern. The cumulative release rate during the first 12 h was 60.3%, and the residue had been released over a longer incubation time.

### Enhancement of the solubility and stability of Que via incorporation of Que into mAb GAP43-Exo

**Fig. 3:**
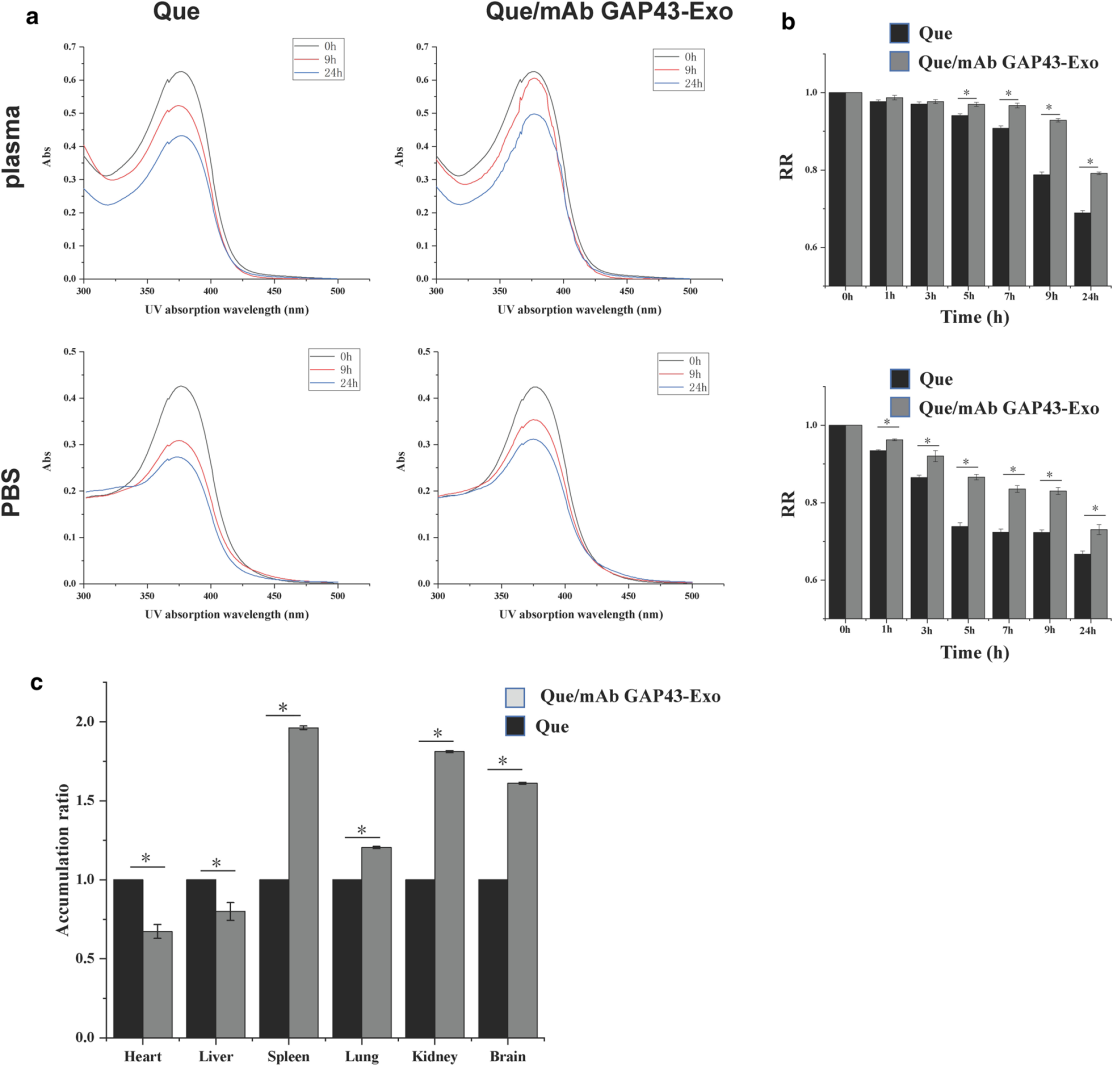
Que/mAb GAP43-Exo enhanced the stability and solubility of Que in vitro. **a** The UV absorption curve of Que and exosomal Que in PBS and plasma. **b** The remaining ratio (RR) of Que and exosomal Que in PBS and plasma. Data are expressed as means ± SD (n = 3), *P < 0.05. **c** Tissue distributions of Que in rats treated with Que and Que/mAb GAP43-Exo at 8 h after single i.v. administration. The accumulation ratio was quantified by comparing the concentrations of exosomal Que in different organs with the concentration of free Que in organs. The accumulation ratio of free Que in organs was set as 1.0. Data are expressed as means ± SD (n = 3), *P < 0.05

After Que was loaded into Exo, the water solubility of Que was significantly enhanced by 2.87-fold as compared to that of free Que (30.15 μg/mL versus 86.97 μg/mL, respectively). This result indicated that the solubility of Que was significantly enhanced by loading into Exo. We detected the UV absorption of free Que and Que/mAb GAP43-Exo at wavelength from 300 to 500 nm, and The UV absorption curve of Que and exosomal Que in PBS and plasma was shown in Fig. 3a. As shown in Fig. 3a, b, free Que was degraded faster than Que/mAb GAP43-Exo and about 33% and 31% of free Que in PBS and plasma respectively, were degraded rapidly after 24 h of dispersion in PBS and plasma. In contrast, when Que was loaded in mAb GAP43-Exo, its stability was significantly enhanced, and it was protected from degradation. The relative RRs of exosomal Que in PBS and plasma had increased significantly to 73% and 79% after 24-h incubation in PBS and plasma, inferring that Que/mAb GAP43-Exo significantly improved the stability of Que by slowing down the oxidative degradation of Que as compared with that of free Que. These results confirmed that our drug delivery system can significantly improve the stability of Que. The instability of Que was mainly due to the oxidative decarboxylation of cyclic C, and Exo as drug carrier reduced the contact between Que and the solution, thus slowing down its oxidative degradation.

### Analysis of drug biodistribution in vivo

We also evaluated the biodistribution of Que and Que/mAb GAP43-Exo in main organs by measuring the accumulation of Que. As shown in Fig. 3c, it demonstrated that compared with free Que, Que/mAb GAP43-Exo reduced the accumulation of Que in the liver and heart, and enhanced its retention in the spleen, lung, kidney, and brain. In particular, the amount of exosomal Que accumulated in the brain was increased to 1.7 times higher than that of free Que, indicating that Que/mAb GAP43-Exo showed a specific brain-targeting ability and that this ability facilitated the transport of Que across the BBB. In addition, Que/mAb GAP43-Exo may lower the metabolism of Que in the liver.

### Que/mAb GAP43-Exo effectively enhanced targeted delivery of Que to GAP43 overexpressed impaired neurons in ischemic areas in vitro and in vivo

**Fig. 4:**
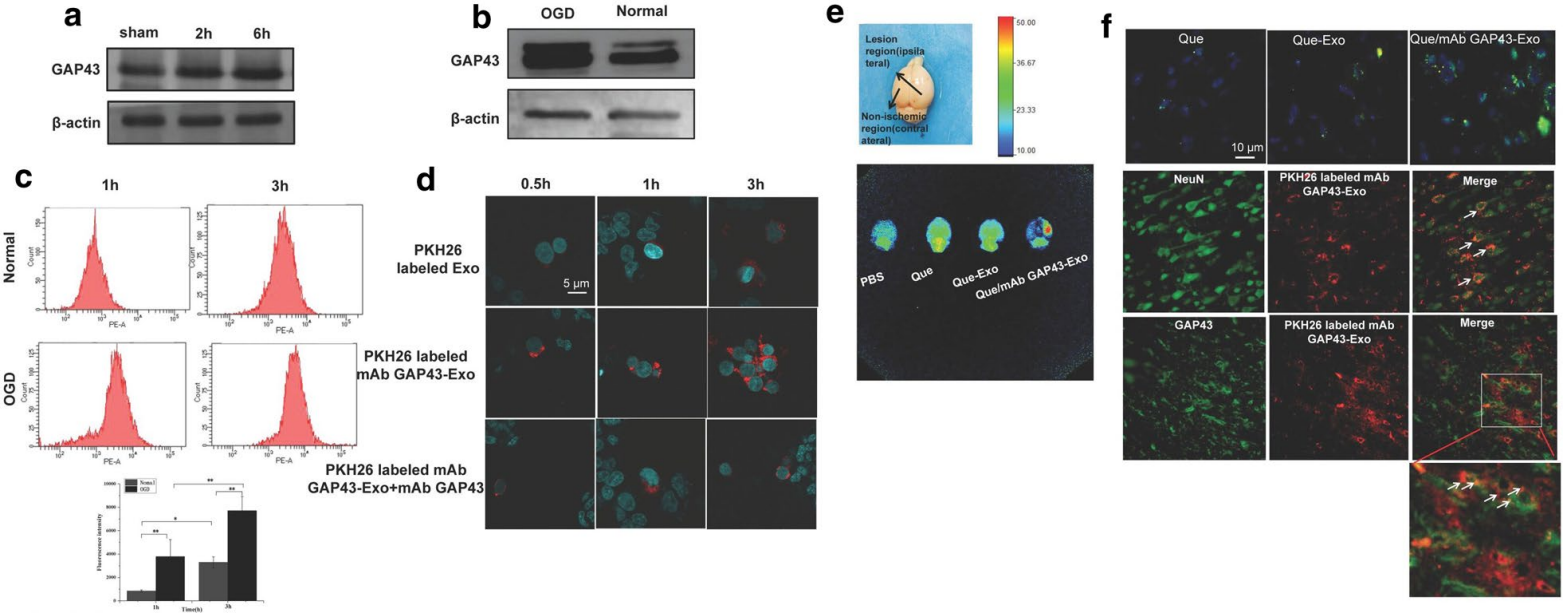
GAP43 mediated special targeting of Que/mAb GAP43-Exo in damaged neurons in vivo and in vitro. **a** Expression of GAP43 in ischemic region of brain at 2 h and 6 h of MCAO/R. **b** Expression of GAP43 in normal SH-SY5Y cells and OGD treated SH-SY5Y cells by western blot. **c** The uptake of PKH26-labeled mAb GAP43-Exo in normal SH-SY5Y cells and OGD treated SH-SY5Y cells by flow cytometry. Data are expressed as means ± SD (n = 3), *P < 0.05, **P < 0.01. **d** Confocal images of OGD treated SH-SY5Y cells after incubating with Exo and mAb GAP43-Exo. Nucleus was stained with Hoechst 33342 (blue) for 15 min at 37 ℃ and all of Exo and mAb GAP43-Exo were labeled by PKH26 (red). The scale bar is 5 μm and applies to all figure parts. **e** Representative fluorescence images of MCAO/R rats brains treated with PBS, Que, Que-Exo and Que/mAb GAP43-Exo via intravenous injection. **f** Location of Que (green) in ischemic brain tissue after treated with Que, Que-Exo and Que/mAb GAP43-Exo. Nucleus was stained with DAPI (blue) for 15 min at 37 ℃. Co-location of PKH26 labeled mAb GAP43-Exo (red) with neurons (green) in ischemic brain tissue. Neuron was stained with NeuN antibodies for 15 min at 37 ℃. Co-location of PKH26 labeled mAb GAP43-Exo (red) with GAP43 (green) in ischemic brain tissue. The scale bar is 10 μm

To assess GAP43 mediated neuronal targeting of Que, SH-SY5Y cells were subjected to OGD to generate ischemia-like neurons. The expressions of GAP43 in SH-SY5Y and OGD-treated SH-SY5Y cells were investigated by Western blotting, and the uptake of PKH26-labelled mAb GAP43-Exo in both cells was also determined by flow cytometry. The results in Fig. 4b demonstrated that the expression of GAP43 was significantly enhanced in OGD-treated cells as compared to normal cells, indicating that SH-SY5Y cells were seriously damaged after OGD, and that the protein expression of GAP43 in OGD-treated SH-SY5Y cells were indeed increased. In addition, more mAb GAP43-Exo was taken up by OGD-treated SH-SY5Y cells than in normal cells, indicating that damaged neurons were specifically targeted in a GAP43-mediated manner (Fig. 4c). To confirm the role of GAP43 in improving the targeting of Exo to damaged neurons, the cellular distributions of PKH26-labeled Exo and PKH26-labeled mAb GAP43-Exo in OGD-treated SH-SY5Y cells were observed by confocal laser scanning microscopy. As shown in Fig. 4d, more PKH26-labeled mAb GAP43-Exo was located in the cytoplasm of OGD-treated SH-SY5Y cells expressing high levels of GAP43 and emitted stronger red fluorescence than PKH26-labeled Exo. This indicated that mAb GAP43-Exo effectively promoted drug accumulation in those OGD-treated cells expressing high levels of GAP43; specific binding between mAb GAP43 and GAP43 might facilitate the uptake of Exo in OGD-treated SH-SY5Y cells. To further clarify the role of GAP43 in mediating the internalization of Exo, cells were preincubated with free mAb GAP43 to block GAP43 in cells, and then mAb GAP43-Exo was added. The results showed that as free mAb GAP43 selectively competed with mAb GAP43 on mAb GAP43-Exo to bind GAP43 expressed on cells, the uptake of mAb GAP43-Exo into cells was significantly inhibited owing to receptor saturation. Therefore, intracellular fluorescence intensity of PKH26-labeled mAb GAP43-Exo was significantly reduced. It indicated that mAb GAP43 may mediate the internalization of Exo into GAP43-overexpressed neurons.

We further examined the brain-targeting ability of Que/mAb GAP43-Exo in MCAO/R rats. Western blotting revealed that the protein expression of the GAP43 in the ischemic penumbra gradually increased after 2 h and 6 h of ischemia/reperfusion when compared with the sham group (Fig. 4a), indicating that after ischemia/reperfusion occurred, more GAP43 proteins accumulated in the ischemic penumbra, which was consistent with the high expression of GAP43 in OGD-treated cells. The results shown in Fig. 4e illustrated that free Que and Que-Exo administrations resulted in weaker fluorescence in the ischemic region than Que/mAb GAP43-Exo administration, indicating that mAb GAP43-Exo increased the amount of Que that crossed the BBB and localized in the ischemic penumbra. The results (Fig. 4f) also showed that the amount of Que delivered to the ischemic region was increased when it was loaded in mAb GAP43-Exo, suggesting that Que/mAb GAP43-Exo was successfully transported to the ischemic region. To further assess the ability of mAb GAP43-Exo to target damaged neurons in the ischemic region, immunostaining for GAP43 and neurons was performed, and mAb GAP43-Exo was labelled with PKH26. PKH26-labelled mAb GAP43-Exo colocalized well with NeuN positive neurons and GAP43 in the ischemic region. This result suggested that PKH26-labelled mAb GAP43-Exo could combine with GAP43 and be internalized into neurons in the ischemic region. Taken together, these data proved that conjugation of mAb GAP43 to Exo helped Exo cross the BBB and improved the targeting of Que to damaged neurons in the ischemic brain, providing a further theoretical basis for the therapeutic utility of Que/mAb GAP43-Exo.

### Que/mAb GAP43-Exo reduced OGD-induced neuronal injury by inhibiting ROS production via activation of the Nrf2/HO-1 pathway

**Fig. 5:**
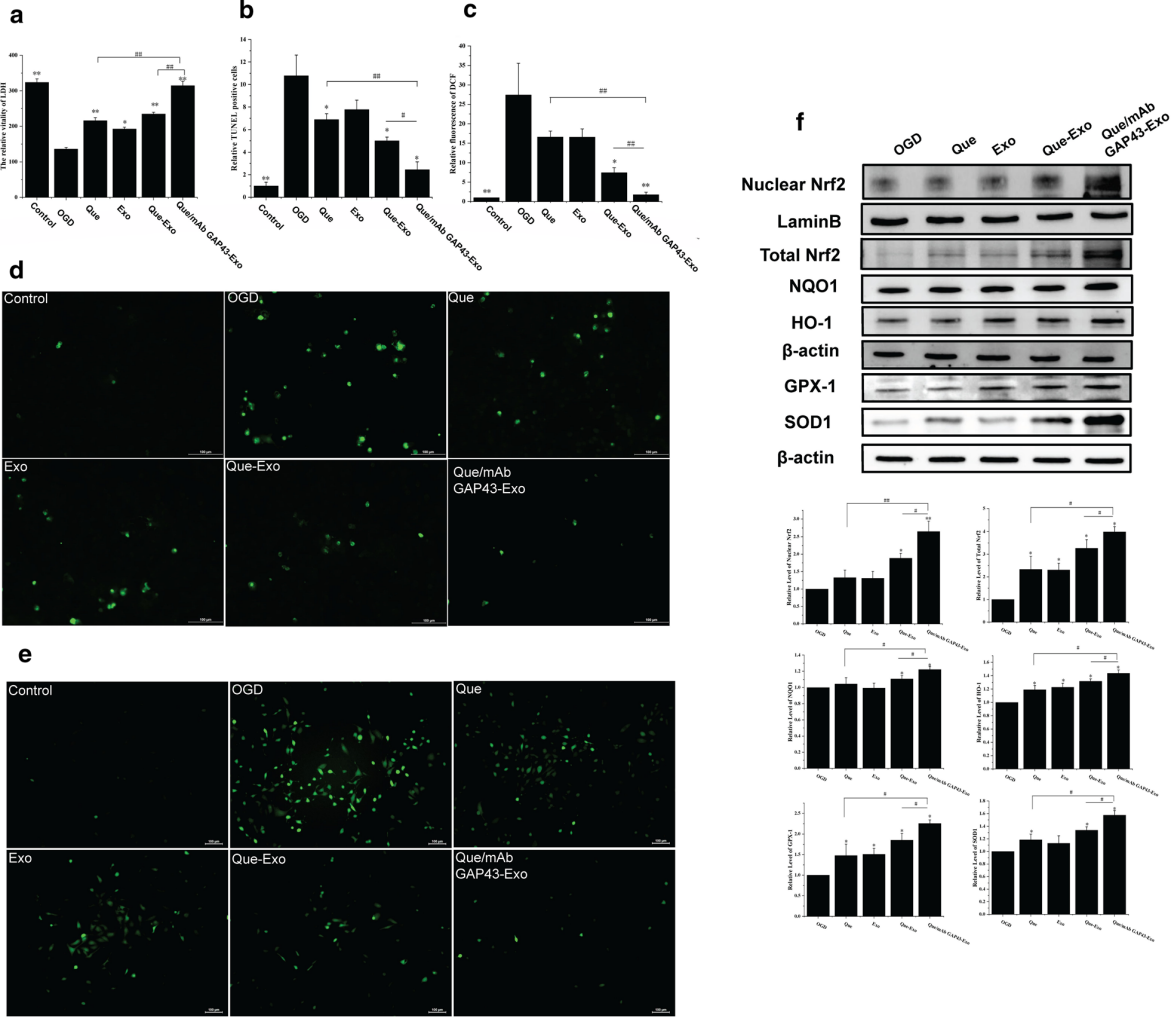
Que/mAb GAP43-Exo reduced OGD mediated neuronal injury in vivo. Normal cells were used as the control group. **a** The effect of LDH in OGD‑induced cells treated with Que, Exo, Que-Exo and Que/mAb GAP43-Exo. Data are expressed as means ± SD (n = 3). *P < 0.05, **P < 0.01, comparing with OGD group, ^##^P < 0.01. **b** Relative ratio of TUNEL positive number of OGD cells treated with Que, Exo, Que-Exo and Que/mAb GAP43-Exo. Relative ratio of TUNEL positive number in control group was set as 1.0. Data represent means ± SD (n = 3), *P < 0.05, **P < 0.01, comparing with OGD group, ^#^P < 0.05, ^##^P < 0.01. **c** Quantitative relative fluorescence assay of DCF in OGD cells treated with Que, Exo, Que-Exo and Que/mAb GAP43-Exo. Quantitative relative fluorescence density of DCF in control group was set as 1.0. Data represent means ± SD (n = 3), *P < 0.05, **P < 0.01, comparing with OGD group, ^##^P < 0.01. **d** Images of apoptotic cells in OGD cells treated with Que, Exo, Que-Exo and Que/mAb GAP43-Exo in TUNEL assay. The scale bar is 100 μm. **e** Images of ROS generation in OGD cells treated with Que, Exo, Que-Exo and Que/mAb GAP43-Exo. The scale bar is 100 μm. **f** Western blot and quantitative analysis of the expression levels of Nrf2, NQO-1, HO-1, SOD1 and GPx1 in OGD cells treated with Que, Exo, Que-Exo and Que/mAb GAP43-Exo. Relative expression of protein in OGD group was set as 1.0. Data are expressed as means ± SD (n = 3). *P < 0.05, **P < 0.01, comparing with OGD group, ^#^P < 0.05, ^##^P < 0.01

As the survival of neurons in the penumbra is neuroprotective in ischemic stroke, we first determined whether Que/mAb GAP43-Exo exerted neuroprotective effects against OGD-induced cell injury by performing LDH assay and TUNEL assay. The LDH results (Fig. 5a) showed that OGD-treated SH-SY5Y cells were severely damaged, exhibiting decreased LDH content compared to control cells. Treatment with Que, Exo, Que-Exo and Que/mAb GAP43-Exo increased cell viability by restoring the activity of LDH. In particular, the Que/mAb GAP43-Exo treated group had the highest LDH activity (Fig. 5a), indicating that Que/mAb GAP43-Exo pretreatment prevented the OGD‑induced reduction in cell viability and significantly enhanced LDH activity. Analysis of TUNEL assay results (Fig. 5b, d) also revealed that following Que/mAb GAP43-Exo treatment, the number of apoptotic nuclei was significantly decreased in OGD-treated SH-SY5Y cells compared with the blank OGD-treated group, indicating that Que/mAb GAP43-Exo attenuated the damage to OGD treated cells. Furthermore, we investigated whether ROS served as a critical regulator and mediated Que/mAb GAP43-Exo-induced increase in cell survival. The results in Fig. 5c, e showed that compared with the control group, OGD-treated SH-SY5Y cells exhibited stronger green fluorescence intensity, indicating that the ROS content was higher in OGD-treated SH-SY5Y cells. After treatment with Que, Que-Exo, Exo and Que/mAb GAP43-Exo, ROS generation was reduced. In particular, compared with the other treatments, Que/mAb GAP43-Exo led to a significant decrease in the ROS content. These results further confirmed that Que/mAb GAP43-Exo exerted antioxidative effects. These results proved that Que/mAb GAP43-Exo can prevent OGD-mediated ROS production, thereby exerting a promising protective effect.

The Nrf2-related pathway has been recognized as a molecular target for ischemic stroke intervention. During the cell oxidative stress response, Nrf2 dissociates from Kelch-like ECH-associated protein 1 (Keap1) and translocates into the nucleus to bind with the antioxidant response element (ARE) to recognize and regulate the downstream gene expressions such as NQO1 and HO-1, thereby reducing various oxidative stress reactions and promoting the restoration of the homeostasis of redox. To test whether Que/mAb GAP43-Exo played a therapeutic role in scavenging ROS through the Nrf2 antioxidant pathway, we performed western blotting to analyze the protein expression levels of Nrf2, NQO-1 and HO-1. The experimental results in Fig. 5f showed that treatment with Que, Que-Exo, Exo, and Que/mAb GAP43-Exo increased the total-Nrf2 expression and further increased the expression of nuclear-Nrf2 in OGD-treated SH-SY5Y cells. Furthermore, they upregulated the NQO-1 and HO-1 levels in OGD-treated SH-SY5Y cells Interestingly, when compared with other treatments, Que/mAb GAP43-Exo significantly promoted the translocation of Nrf2 into the nucleus, showing the highest nuclear Nrf2 levels and increased levels of NQO-1 and HO-1. These results showed that Que/mAb GAP43-Exo treatment activated the Nrf2 pathway by increasing the protein expressions of Nrf2, NQO-1, and HO-1. Meanwhile, when OGD-treated SH-SY5Y cells were treated with Que, Que-Exo, Exo and Que/mAb GAP43-Exo, superoxide dismutase (SOD) and glutathione peroxidase (GPx) were activated. Notably, Que/mAb GAP43-Exo resulted in a significant increase in SOD1 and GPx1 activities as compared to the other groups. Taken together, these data confirmed that Que/mAb GAP43-Exo exerted neuroprotective effects by regulating ROS, contributed to the activation of the Nrf2/HO-1 pathway and resulted in the suppression of ROS in OGD-treated SH-SY5Y cells.

### Que/mAb GAP43-Exo contributed to potential neuroprotection by effectively reducing neuronal injury in the MCAO/R model

**Fig. 6:**
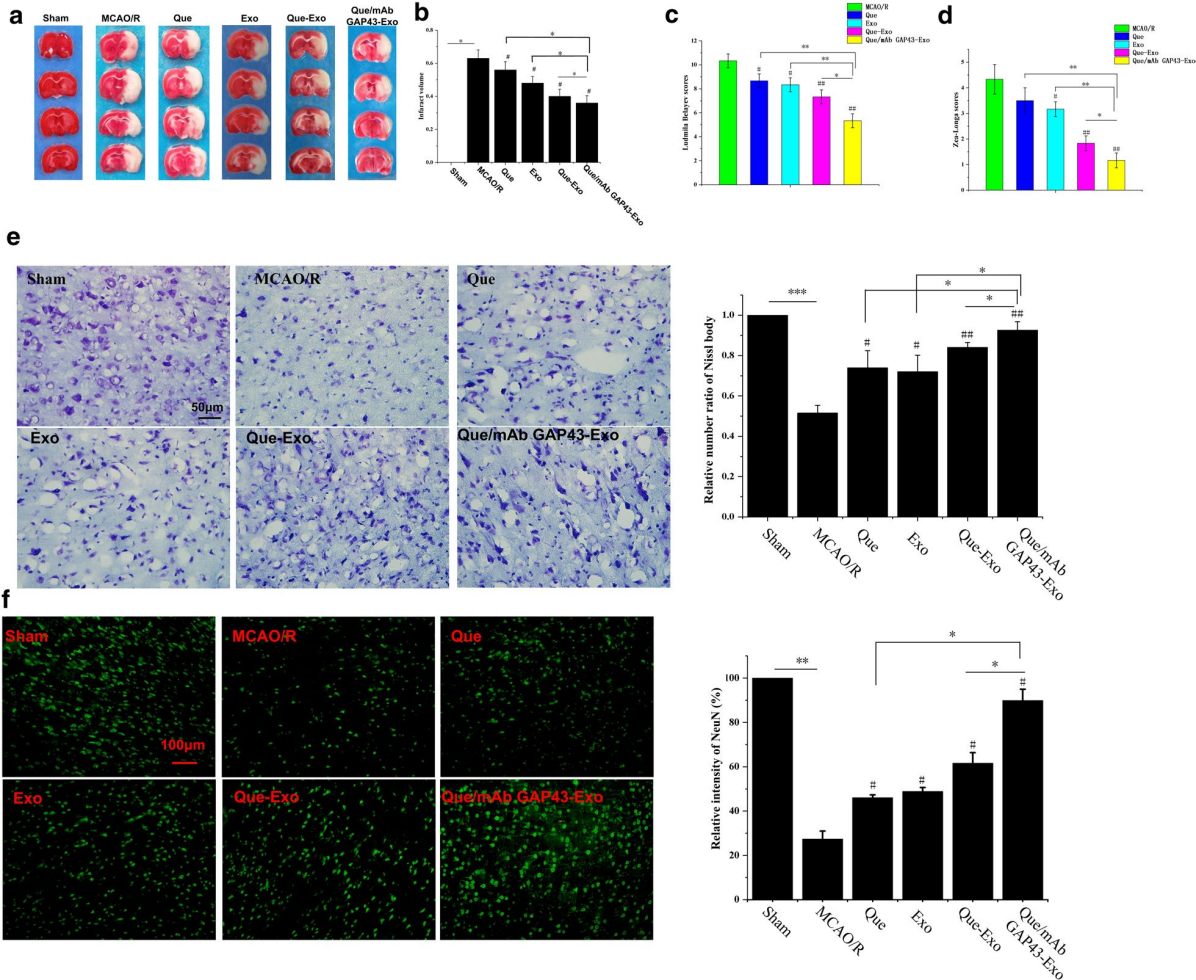
Neuroprotective role of Que/mAb GAP43-Exo at 2 h of ischemia following by 24 h reperfusion on MCAO/R rats. **a** Representative brain slices with infarcts stained by 2,3,5-triphenyltetrazolium chloride (TTC) from each group at 2 h of ischemia following by 24 h reperfusion on MCAO/R rats. **b** Infarct volume on MCAO/R model treated with PBS, Que, Exo, Que-Exo and Que/mAb GAP43-Exo. Data are expressed as means ± SD (n = 3). ^#^P < 0.05, comparing with infarct volume on MCAO/R model, *P < 0.05. **c** Ludmila Belayev neurological scores. Data are expressed as means ± SD (n = 3). ^#^P < 0.05, ^##^P < 0.01, comparing score on MCAO/R model. *P < 0.05, **P < 0.01. **d** Zea-Longa neurological scores. Data are expressed as means ± SD (n = 3). ^#^P < 0.05, ^##^P < 0.01, comparing score on MCAO/R model. *P < 0.05, **P < 0.01. **e** Nissl staining and quantitative analysis in MCAO/R models treated with PBS, Que, Exo, Que-Exo and Que/mAb GAP43-Exo. The relative number ratio of Nissl body in sham group was set as 1.0. Data are expressed as means ± SD (n = 3), ^#^P < 0.05, ^##^P < 0.01, comparing with MCAO/R model group. *P < 0.05, ***P < 0.001. The scale bar is 50 μm. **f** Representative immunofluorescence staining and quantitative analysis for neurons. Relative fluorescence intensity of NeuN in sham group was set as 100%. NeuN antibodies were used to stain neurons in the ischemic penumbra. Data are expressed as means ± SD (n = 3), ^#^P < 0.05, comparing with MCAO/R model group. *P < 0.05, **P < 0.01. The scale bar is 100 μm

The neuroprotective effects of Que and Que/mAb GAP43-Exo were investigated in the MCAO/R model. After 2 h of ischemia followed by 24 h of reperfusion, the brains of MCAO/R rats treated with Que, Exo, Que-Exo and Que/mAb GAP43-Exo were subjected to TTC staining, and the cerebral infarct volume was calculated. Figures 6a, b showed that the cerebral infarct volume was reduced in Que, Exo, Que/Exo and Que/mAb GAP43-Exo treated groups as compared to PBS treated MCAO/R group. Among these treatments, Que/mAb GAP43-Exo showed the best neuroprotective effects, as the cerebral infarction volume was the smallest. Rats in each group were subjected to Zea-Longa and Ludmila Belayev scores to evaluate the degree of neurological improvement in MCAO/R rats (Fig. 6c, d). The results showed that Que/mAb GAP43-Exo treatment significantly improved neurological function. Compared with other groups, Zea-Longa and Ludmila Belayev scores in the Que/mAb GAP43-Exo treated group were significantly reduced, and the difference was statistically significant (*P* < 0.05).

As the neuronal damage contributed to poor outcomes related to recovery after cerebral ischemia/reperfusion injury, Que/mAb GAP43-Exo mediated increase in neuronal survival in the ischemic penumbra was further investigated. After 2 h of ischemia followed by 24-h reperfusion, rats were sacrificed and the degree of neuronal degeneration and necrosis in the ischemic area of the brain were assessed by Nissl staining. The results presented in Fig. 6e showed that after ischemia/reperfusion, the cell bodies of neurons were expanded and irregularly arranged, and few Nissl bodies were observed, indicating that ischemia/reperfusion induced neuronal damage by altering the biological structure of neurons. After treatment with Que, Exo, Que-Exo, and Que/mAb GAP43-Exo, the number of Nissl bodies was slightly increased. Moreover, in the Que/mAb GAP43-Exo treated group, the number of Nissl bodies was higher than that in the other groups, and most neurons maintained their structural integrity. The immunofluorescence staining results (Fig. 6f) demonstrated that when compared with few NeuN positive cells found in the Que, Exo, and Que-Exo treated groups, more NeuN positive cells were observed in the Que/mAb GAP43-Exo treated group. This result suggested that Que/mAb GAP43-Exo exerted a significant neuroprotective effect by reducing the cerebral infarct area and improving neurological function in MCAO/R rats. Furthermore, Que/mAb GAP43-Exo potentially alleviated neuronal injury after ischemia/reperfusion and exerted synergistic therapeutic effects.

### Que/mAb GAP43-Exo treatment reduced ROS production by activating the Nrf2/HO-1 pathway in the MCAO/R model

**Fig. 7:**
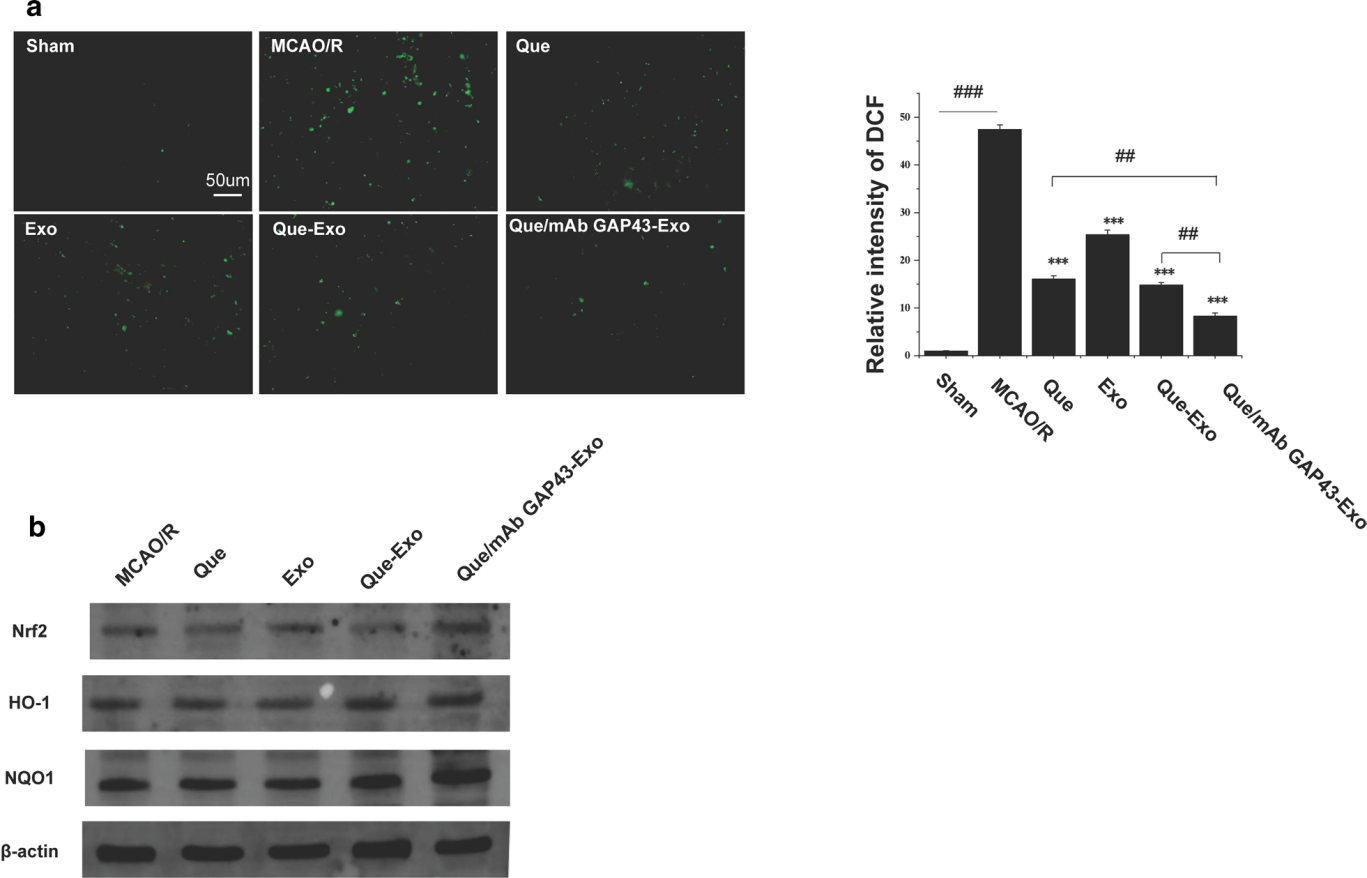
Que/mAb GAP43-Exo inhibited generation of ROS by activating the Nrf2/HO-1 pathway. **a** ROS generation in ischemia/reperfusion brain tissue treated with PBS, Que, Exo, Que-Exo and Que/mAb GAP43-Exo. Relative fluorescence density of DCF in sham group was set as 1.0. Data are expressed as means ± SD (n = 3), ***P < 0.001, comparing with MCAO/R group, ^##^P < 0.01, ^###^P < 0.001. The scale bar is 50 μm. **b** Western blot of the expression levels of Nrf2, NQO-1 and HO-1 in ischemia/reperfusion brain tissue

After MCAO/R, a series of oxidative stress reactions resulting from the production of a large amount of ROS occurred in brain tissue, causing aggravation of neuronal apoptosis in the ischemic penumbra and exacerbation of infarction. We first studied Que/mAb GAP43-Exo-induced ROS inhibition and explored the potential molecular mechanism in rats after MCAO/R. The amount of ROS in the ischemic penumbra was quantified. As shown in Fig. 7a, when compared with other groups, Que/mAb GAP43-Exo significantly suppressed the generation of ROS. We also analyzed the expression levels of proteins such as Nrf2, NQO-1 and HO-1 in ischemic brain tissue. The experimental results in Fig. 7b showed that when compared with the MCAO/R group, levels of Nrf2, NQO-1, and HO-1 were upregulated after treatment with Que, Que-Exo, Exo and Que/mAb GAP43-Exo. Interestingly, Que/mAb GAP43-Exo had the highest expression levels of Nrf2, NQO-1, and HO-1 in the ischemic penumbra. This result illustrated that Que/mAb GAP43-Exo treatment induced the activation of the Nrf2/HO-1 pathway. Que/mAb GAP43-Exo can increase the expressions of antioxidant proteins in the ischemic penumbra to clear ROS produced in the area, thereby exerting a protective effect in the ischemic penumbra.

## Discussion

Oxidative stress is considered as an important contributor to neurodegenerative ischaemia/reperfusion injury. When excessive ROS are generated or insufficiently neutralized, they accumulate in the cell and then trigger oxidative stress, which further causes cell damage and ischemic infarction. The effect of Que depends on its unique molecular structure to scavenge ROS, and its antioxidant capacity contributes to its neuroprotective effects through regulation of the antioxidant defense of neurons. However, Que is difficult to dissolve in water, resulting in limited absorption, poor stability and an inability to cross biological barriers.

In this study, we chose plasma exosomes as multifunctional biomimetic drug delivery vehicles to improve the stability and brain-targeting abilities of Que. The results demonstrated that compared with those of free Que, the stability and solubility of exosomal Que were significantly enhanced. Consider that GAP43 is highly expressed in damaged neurons, we conjugated mAb GAP43 to the surface of Exo to allow an interaction between Exo and GAP43 expressed on neurons in the ischemic region. It was found that mAb GAP43-Exo could be efficiently internalized into OGD-treated SH-SY5Y cells expressing higher levels of GAP43 duo to the interactions between mAb GAP43-Exo and GAP43 expressed on cells. Moreover, mAb GAP43-Exo increased drug penetration across the BBB and promoted delivery of Que into damaged neurons with high GAP43 expression in MCAO/R rats.

We also investigated the neuroprotective effects of Que/mAb GAP43-Exo against ischemia/reperfusion injury in vitro and in vivo, and revealed the potential mechanism. The LDH results showed that Que/mAb GAP43-Exo not only improved the survival rate of SH-SY5Y cells after OGD but also significantly inhibited the apoptosis of neurons in MCAO/R rats by improving the morphology of Nissl bodies and increasing the number of NeuN positive cells. Finally, Que/mAb GAP43-Exo significantly alleviated ischemic damage in the brain tissue by reducing the infarct area and improving overall neurological performance.

To confirm the antioxidative effects of Que/mAb GAP43-Exo, the production of ROS was evaluated in OGD-treated SH-SY5Y cells and in MCAO/R-injured rats. The ROS content was markedly decreased in OGD treated SH-SY5Y cells following treatment with Que/mAb GAP43-Exo, and ROS levels in the ischemic penumbra area was significantly reduced.

As previous studies had confirmed that as Que can prevent oxidative stress-induced cell injury via the activation of the Nrf2/HO-1 pathway, we explored the role of Que/mAb GAP43-Exo in regulating the Nrf2/HO-1 pathway. The expression levels of Nrf2 and its downstream proteins NQO-1 and HO-1 were significantly increased in the Que/mAb GAP43-Exo-treated group compared to the other treated groups. This result indicated that Que/mAb GAP43-Exo administration induced the activation of the Nrf2/HO-1 pathway, thus reducing the massive accumulation of ROS and alleviating neurological deficits.

We also knew that Exo exerted its own neuroprotective effects in the treatment of ischemic stroke. In our previous study, we had found that Exo suppressed neuronal cell apoptosis and alleviated BBB damage, thus inducing neuroprotection and functional improvement after ischemic stroke [31]. In this study, we also confirmed that naïve Exo exerted a synergistic neuroprotective effect against ischemia/reperfusion injury.

## Conclusion

We successfully prepared a damaged neuron-targeting, quercetin-loaded, monoclonal antibody against GAP43-decorated plasma exosomes to alleviate ischemia/reperfusion-induced injury. This study provides strong evidence that Que/mAb GAP43-Exo enhanced the accumulation of Que in the ischemic brain through the mAb GAP43-mediated targeting of drugs to impaired neurons. Moreover, it decreased the production of ROS, as well as increased the level of LDH. Notably, Que/mAb GAP43-Exo attenuated oxidative stress-induced ischemia/reperfusion injury and induced the activation of the Nrf2/HO-1 pathway by promoting the nuclear translocation of Nrf2 and upregulating expressions of NQO-1, HO-1, SOD1 and GPx1. Taken together, these results suggested that Que/mAb GAP43-Exo could be a potential effective remediation strategy in ischemia/reperfusion injury therapeutics.

## Supplementary Information

**Additional file 1.** In vitro release profile of free-Que and Que/mAb GAP43-Exo in release medium (PBS, pH 7.4, 37°C) containing SDS for 12 h. Data are expressed as means ± SD (n=3)

## Data Availability

All data generated or analyzed during this study are included in this published article.

## Abbreviations

Que: Quercetin
GAP43: Growth-associated protein-43
BBB: Blood–brain barrier
MCAO/R: Middle cerebral artery occlusion /reperfusion
ROS: Reactive oxygen species

## References

[1] Mozaffarian D, Benjamin EJ, Go AS (2015). Heart disease and stroke statistics–2015 update: a report from the American Heart Association. Circulation.

[2] Virani SS, Alonso A, Benjamin EJ (2020). Heart disease and stroke statistics-2020 update: a report from the American Heart Association. Circulation.

[3] Tobin MK, Bonds JA, Minshall RD (2014). Neurogenesis and inflammation after ischemic stroke: what is known and where we go from here. J Cerebral Blood Flow Metabol Official.

[4] O'Collins VE, Macleod MR, Donnan GA (2006). 1,026 experimental treatments in acute stroke. Ann Neurol.

[5] Pereira AC, Martin PJ, Warburton EA (2001). Thrombolysis in acute ischaemic stroke. Postgrad Med J.

[6] Yu J, Wang WN, Matei N (2020). Ezetimibe attenuates oxidative stress and neuroinflammation via the AMPK/Nrf2/TXNIP pathway after MCAO in rats. Oxid Med Cell Longev.

[7] Rivera F, Costa G, Abin A (2008). Reduction of ischemic brain damage and increase of glutathione by a liposomal preparation of quercetin in permanent focal ischemia in rats. Neurotox Res.

[8] Kaspar JW, Niture SK, Jaiswal AK (2009). Nrf 2: INrf2 (Keap1) signaling in oxidative stress. Free Radic Biol Med.

[9] He CH, Gong P, Hu B (2001). Identification of activating transcription factor 4 (ATF4) as an Nrf2-interacting protein. Implication for hemeoxygenase-1 gene regulation. J Biol Chem..

[10] Li L, Liu T, Liu L (2019). Effect of hydrogen-rich water on the Nrf2/ARE signaling pathway in rats with myocardial ischemia-reperfusion injury. J Bioenerg Biomembr.

[11] Wu J, Jin Z, Yang X (2018). Post-ischemic administration of 5-methoxyindole-2-carboxylic acid at the onset of reperfusion affords neuroprotection against stroke injury by preserving mitochondrial function and attenuating oxidative stress. Biochem Biophys Res Commun.

[12] Ahmad A, Khan MM, Hoda MN (2011). Quercetin protects against oxidative stress associated damages in a rat model of transient focal cerebral ischemia and reperfusion. Neurochem Res.

[13] Zhang ZJ, Cheang LC, Wang MW (2011). Quercetin exerts a neuroprotective effect through inhibition of the iNOS/NO system and pro-inflammation gene expression in PC12 cells and in zebrafish. Int J Mol Med.

[14] Li C, Zhang WJ, Frei B (2016). Quercetin inhibits LPS-induced adhesion molecule expression and oxidant production in human aortic endothelial cells by p38-mediated Nrf2 activation and antioxidant enzyme induction. Redox Biol.

[15] Lee YJ, Bernstock JD, Nagaraja N (2016). Global SUMOylation facilitates the multimodal neuroprotection afforded by quercetin against the deleterious effects of oxygen/glucose deprivation and the restoration of oxygen/glucose. J Neurochem.

[16] Suganthy N, Devi KP, Nabavi SF, et al. Bioactive effects of quercetin in the central nervous system: Focusing on the mechanisms of actions. Biomed Pharmacother. 2016; 84:892–908. 10.1016/j.biopha.2016.10.011 27756054

[17] Song H, Su C, Cui W (2013). Folic acid-chitosan conjugated nanoparticles for improving tumor-targeted drug delivery. Biomed Res Int..

[18] Zhao L, Li H, Shi Y (2014). Nanoparticles inhibit cancer cell invasion and enhance antitumor efficiency by targeted drug delivery via cell surface-related GRP78. Int J Nanomedicine.

[19] Abdelhamid HN, Dowaidar M, Langel Ü (2020). Carbonized chitosan encapsulated hierarchical porous zeolitic imidazolate frameworks nanoparticles for gene delivery. Microporous Mesoporous Mater.

[20] Abdelhamid HN, Dowaidar M, Hällbrink M, Langel Ü (2020). Gene delivery using cell penetrating peptides-zeolitic imidazolate frameworks. icroporous. Mesoporous Mater.

[21] Mehryab F, Rabbani S, Shahhosseini S (2020). Exosomes as a next-generation drug delivery system: an update on drug loading approaches, characterization, and clinical application challenges. Acta Biomater.

[22] Wang Y, Zhang Y, Cai G (2020). Exosomes as actively targeted nanocarriers for cancer therapy. Int J Nanomedicine.

[23] Zheng YZ, Hasan A, Nejadi Babadaei MM (2020). Exosomes: multiple-targeted multifunctional biological nanoparticles in the diagnosis, drug delivery, and imaging of cancer cells. Biomed Pharmacother.

[24] Zou J, Shi M, Liu X (2019). Aptamer-functionalized exosomes: elucidating the cellular uptake mechanism and the potential for cancer-targeted chemotherapy. Anal Chem.

[25] Luo ZW, Li FX, Liu YW (2019). Aptamer-functionalized exosomes from bone marrow stromal cells target bone to promote bone regeneration. Nanoscale.

[26] Tian T, Zhang HX, He CP (2018). Surface functionalized exosomes as targeted drug delivery vehicles for cerebral ischemia therapy. Biomaterials.

[27] Wang CY, Lin HC, Song YP (2015). Protein kinase C-dependent growth-associated protein 43 phosphorylation regulates gephyrin aggregation at developing GABAergic synapses. Mol Cell Biol.

[28] Gorup D, Bohacek I, Milicevic T (2015). Increased expression and colocalization of GAP43 and CASP3 after brain ischemic lesion in mouse. Neurosci Lett.

[29] Wang AR, Hu MZ, Zhang ZL (2019). Fastigial nucleus electrostimulation promotes axonal regeneration after experimental stroke via cAMP/PKA pathway. Neurosci Lett.

[30] Sandelius A, Cullen NC, Kallen A (2018). Transient increase in CSF GAP43 concentration after ischemic stroke. BMC Neurol.

[31] Jiang Y, He R, Shi Y (2020). Plasma Exosomes protect against cerebral ischemia/reperfusion injury via Exosomal HSP70 mediated suppression of ROS. Life Sci..

[32] Garcia-Contreras M, Shah SH, Tamayo A (2017). Plasma-derived exosome characterization reveals a distinct microRNA signature in long duration Type 1 diabetes. Sci Rep.

[33] Kalra H, Adda CG, Liem M, et al. Comparative proteomics evaluation of plasma exosome isolation techniques and assessment of the stability of exosomes in normal human blood plasma. Proteomics. 2013; 13:3354–64.

[34] Qi Y, Guo L, Jiang Y (2020). Brain delivery of quercetin-loaded exosomes improved cognitive function in AD mice by inhibiting phosphorylated Tau-mediated neurofibrillary tangles. Drug Deliv.

[35] Wang H, Sui H, Zheng Y (2019). Curcumin-primed exosomes potently ameliorate cognitive function in AD mice by inhibiting hyperphosphorylation of the Tau protein through the AKT/GSK-3β pathway. Nanoscale.

[36] Zhang J, Ding C, Zhang S (2020). Neuroprotective effects of astaxanthin against oxygen and glucose deprivation damage via the PI3K/Akt/GSK3β/Nrf2 signalling pathway in vitro. J Cell Mol Med.

[37] Jin W, Xu W, Zhang X (2020). Ischemic preconditioning upregulates decoy receptors to protect SH-SY5Y cells from OGD induced cellular damage by inhibiting TRAIL pathway and agitating PI3K/Akt pathway. Mol Neurobiol.

[38] Théry C, Witwer KW, Aikawa E (2018). Minimal information for studies of extracellular vesicles 2018 (MISEV2018): a position statement of the International Society for Extracellular Vesicles and update of the MISEV2014 guidelines. J Extracell Vesicles.

