# Supplementary figures and images for: Surface-modified engineered exosomes attenuated cerebral ischemia/reperfusion injury by targeting the delivery of quercetin towards impaired neurons
Source: J Nanobiotechnology. 2021 May 17;19:141. doi: 10.1186/s12951-021-00879-4 (PMC8130330; doi:10.1186/s12951-021-00879-4)

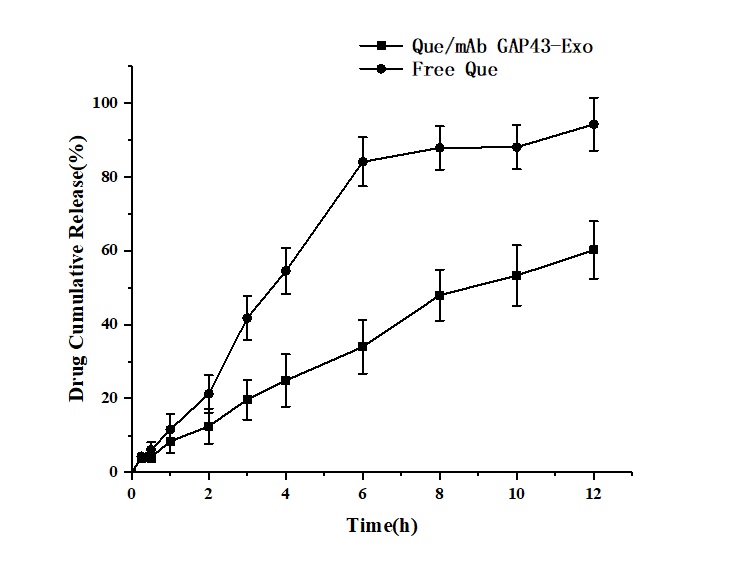

Supplement: Supplementary file 1 — Additional file 1. In vitro release profile of free-Que and Que/mAb GAP43-Exo in release medium (PBS, pH 7.4, 37°C) containing SDS for 12 h. Data are expressed as means ± SD (n=3) [file 12951_2021_879_MOESM1_ESM.jpg]
